# Hyperlipidemia-associated specific modules and hub genes revealed by integrative methods of WGCNA and MetaDE

**DOI:** 10.3389/fgene.2025.1592778

**Published:** 2025-12-10

**Authors:** Zhiyi Zhao, Yin Cao, Anna Gu, Hanxin Yao

**Affiliations:** 1 Department of Andrology, The First Hospital of Jilin University, Jilin, China; 2 Department of Internal Medicine, Northeast Normal University Hospital, Jilin, China; 3 Department of Clinical Laboratory, The First Hospital of Jilin University, Jilin, China

**Keywords:** hyperlipidemia, specific modules, hub genes, weighted gene co-expression network analysis, RAF1, GRK3, CXCR2

## Abstract

**Background and objectives:**

This study aimed to screen the specific modules and hub genes of hyperlipidemia.

**Methods:**

Three microarray datasets (GSE3059, GSE1010 and GSE13985) were obtained from the Gene Expression Omnibus database. Weighted Gene Co-expression Network Analysis (WGCNA) was used to screen stable gene modules across the three datasets. The MetaDE method was used to screen differentially expressed genes (DEGs) based on GSE1010 and GSE13985 datasets. The Cytoscape software was utilized for protein-protein interaction (PPI) network visualization, and the DAVID was employed to perform Kyoto Encyclopedia of Genes and Genomes (KEGG) signaling pathway analysis on the DEG nodes in the interaction network.

**Results:**

Based on the WGCNA algorithm, eleven hyperlipidemia-related modules were screened, and a total of 1,149 DEGs were screened by the metaDE method. 288 overlapping genes were used to establish a PPI network. Finally, a total of four overlapping KEGG pathways were obtained, including Fc gamma R-mediated phagocytosis (*ARPC1A*, *GAB2*, *LYN*, *HCK*, *RAF1*, *WAS*), chemokine signalling pathway (*LYN*, *PTK2B*, *HCK*, *GRK6*, *GRK3*, *RAF1*, *CXCR2*, *JAK3*, *FOXO3*, *WAS*), endocytosis (*CHMP2A*, *CHMP1B*, *RAB5C*, *VPS45*, *TGFBR2*, *GRK6*, *GRK3*, *CXCR2*) and natural killer cell-mediated cytotoxicity (*TNFRSF10C*, *PTK2B*, *ICAM2*, *RAF1*, *FCGR3B*, *SH3BP2*). The hub genes such as *RAF1*, *GRK3* and *CXCR2,* might be potential genetic biomarkers of hyperlipidemia.

**Discussion:**

This study identified the critical pathways and hub genes associated with hyperlipidemia. In particular, these critical pathways may help clarify the pathogenesis of hyperlipidemia, and the hub genes may become new biomarkers and therapeutic target for hyperlipidemia.

## Introduction

Hyperlipidemia refers to the high level of blood fat, which is one of the major risk factors for cardiovascular and cerebrovascular diseases, such as atherosclerosis, coronary heart disease, myocardial infarction and so on (Wang et al., 2019; Piacentini et al., 2020; Ling et al., 2020). Hyperlipidemia is relatively common in middle-aged and elderly people and can induce a variety of cardiovascular diseases. In 2017, 3.9 million people worldwide died of high non-high density-lipoprotein cholesterol. Half of these people were in East, South and Southeast Asia (NCD risk factor collaboration, 2020). In recent years, the incidence of hyperlipidemia has shown an obvious upward trend, and reasonable prevention and regulation are essential.

Recently, the rapid development of high-throughput microarray technology has promoted the recognition of genomic variation and contributed to the in-depth understanding of the pathogenesis of various diseases and the development of promising biomarkers (Liu et al., 2016; Ren et al., 2013; Yan, 2018). The research results of Cesaro et al. demonstrated that PCSK9 was upregulated in patients with hyperlipidemia (Cesaro et al., 2020). These techniques have been used in the study of the potential mechanism of hyperlipidemia. Weighted gene co-expression network analysis (WGCNA) is a common method for constructing gene co-expression networks based on gene expression data (Zhai et al., 2017; Guan et al., 2020).

In a previous study, the GSE3059 dataset has been studied, the hub genes, including *HDAC4*, *F2RL1*, *TMASF1*, *ABHD2* and *FAM13A,* were identified. However, the analysis results of a single dataset have certain limitations (Miao et al., 2018). In this study, three hyperlipidemia microarray datasets were selected and the WGCNA algorithm was used to screen the gene modules with significant stability among multiple datasets. A meta comprehensive algorithm was performed to identify the genes with the consistency difference between the control and hyperlipidemia group. The significant pathways of hyperlipidemia were identified by Gene Ontology (GO) and Kyoto Encyclopedia of Genes and Genomes (KEGG) pathway analysis. These results might aid further investigation of the specific modules and feature genes of hyperlipidemia, as well as provide novel evidence into the underlying genetic biomarkers and signal pathways of treating hyperlipidemia.

## Materials and methods

### Microarray data

Using “Hyperlipidemia, Human” as the keywords, all publicly-uploaded expression profile data were searched in the National Center For Biotechnology Information’s GEO database (http://www.ncbi.nlm.nih.gov/geo/). A total of three datasets, including GSE3059 (n = 32; hyperlipidemia, 32), GSE1010 (n = 24; hyperlipidemia, 12; control, 12) and GSE13985 (n = 10; hyperlipidemia, 5; control, 5), were included in the study. GSE3059 and GSE13985 were based on the platform GPL570 of Affymetrix Human Genome U133 Plus 2.0 Array. GSE1010 was based on the platform GPL96 of Affymetrix Human Genome U133A Array. The screening criteria for datasets were as follows: ① the dataset was gene expression profile data; ② blood samples of hyperlipidemia patients; ③ have clinical information or normal control samples. We downloaded the original expression profile data for further analysis and research.

### Significant gene module based on the WGCNA algorithm

In this study, GSE3059 with the largest sample size was used as the training dataset, whereas GSE1010 and GSE13985 were used for the validation sets. The WGCNA package (version 1.61, https://cran.r-project.org/web/packages/WGCNA/index.html) in R 3.4.1 language was used to screen stable gene modules related to hyperlipidemia (Langfelder and Horvath, 2008). The parameters of WGCNA were set as default values for most general scenarios. Firstly, the genes whose expression coefficient of variation was less than 0.4 were removed; then, the adjacency function based on the training dataset according to the expression correlation between the training and validation datasets was defined, and the gene modules were divided. Then, the coefficient of heterogeneity between genes was calculated, and the systematic clustering tree was obtained (method = average). The screening thresholds for gene module division are as follows: gene module set min size = 150 and cut Height = 0.995. Finally, the stability of the modules across the dataset was calculated with the two verification date sets, and *P*-values <0.05 were considered statistically significant.

### Screening of differentially expressed genes (DEGs)

MetaDE.ES in MetaDE (https://cran.r-project.org/web/packages/MetaDE/) of the R3.4.1 language (Qi et al., 2016; Wang et al., 2012) was used to select DEGs and identifythe consistency of the DEGs among the two datasets (GSE1010 and GSE13985). Firstly, aheterogeneity test was performed on the expression value of each gene under different experimental platforms through the MetaDE.ES analysis method, and the statistical parameters, which include tau^2^, Q value, and Qpval, were obtained to judge the heterogeneity. Then, the differences in expression of the genes in the samples of the integrated dataset in different types of groups were tested to obtain the *P-*value. Therefore, the selection of threshold parameters was as follows: (1) Tau^2^ = 0 and Qpval >0.05 were selected as the homogeneity test parameter to ensure that the source of each selected characteristic gene is homogenous and unbiased; (2) A false discovery rate (FDR) < 0.05 was set as the threshold for statistical significance in expression between groups. The MetaDE thresholds ensured that identified DEGs showed consistent expression directions across multiple datasets, indirectly combining datasets to expand the sample size.

### Construction of protein-protein interaction (PPI) network

The intersection of the genes in the conservative gene module screened by the WGCNA algorithm and the significantly consistent DEGs screened by the MetaDE algorithm were obtained, and the biological process and KEGG signal pathway that is significantly associated with the intersection gene were annotated based on DAVID version 6.8 (https://david.ncifcrf.gov/) (Huang Da et al., 2009a; Huang Da et al., 2009b). String Version 10.5 (https://string-db.org/) was used to investigate the interaction relationship of intersection genes and constructed an interaction network based on this (Szklarczyk et al., 2017). The PPI network was visualised using the Cytoscape software (version 3.6.1, http://www.cytoscape.org/) (Shannon et al., 2003). The KEGG signaling pathways were analyzed in terms of the differential gene nodes in the interaction network based on DAVID.

### Screening of key genes in patients with hyperlipidemia

**FIGURE 1:**
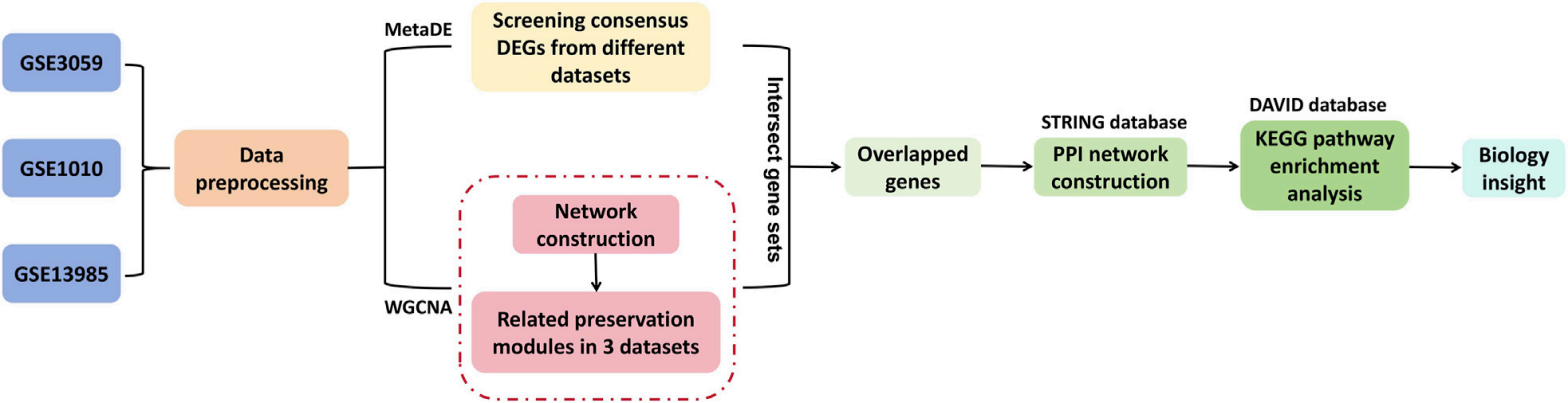
Schematic diagram of the analysis process.

In the Comparative Toxicogenomics Database (CTD database, 2019 http://ctd.mdibl.org/), ‘hyperlipidemia’ was used as the keyword to search for the KEGG pathway directly associated with hyperlipidemia, which was compared with the pathway that was significantly involved in the interaction network, with the aim of constructing the pathway network directly associated with hyperlipidemia and to screen key genes. By searching “hyperlipidemia” as the keyword in the Comparative Toxicogenomics Database (CTD database, 2019 http://ctd.mdibl.org/), KEGG pathway associated with ‘hyperlipidemia’ was obtained. Then, KEGG pathway enrichment analysis was conducted on the genes contained in the interaction network. The KEGG pathway obtained from the two parts was compared, and the overlapped part was preserved. These pathways are directly related to hyperlipidemia and contain key genes that we have screened (Grondin et al., 2018). Figure 1 showed a flowchart of the analysis methods, including the construction of WGCNA, MetaDE, and PPI networks, so as to help understand the process of identifying key genes.

## Results

### Screening of key modules related to disease significance based on meta-integrated WGCNA

**FIGURE 2:**
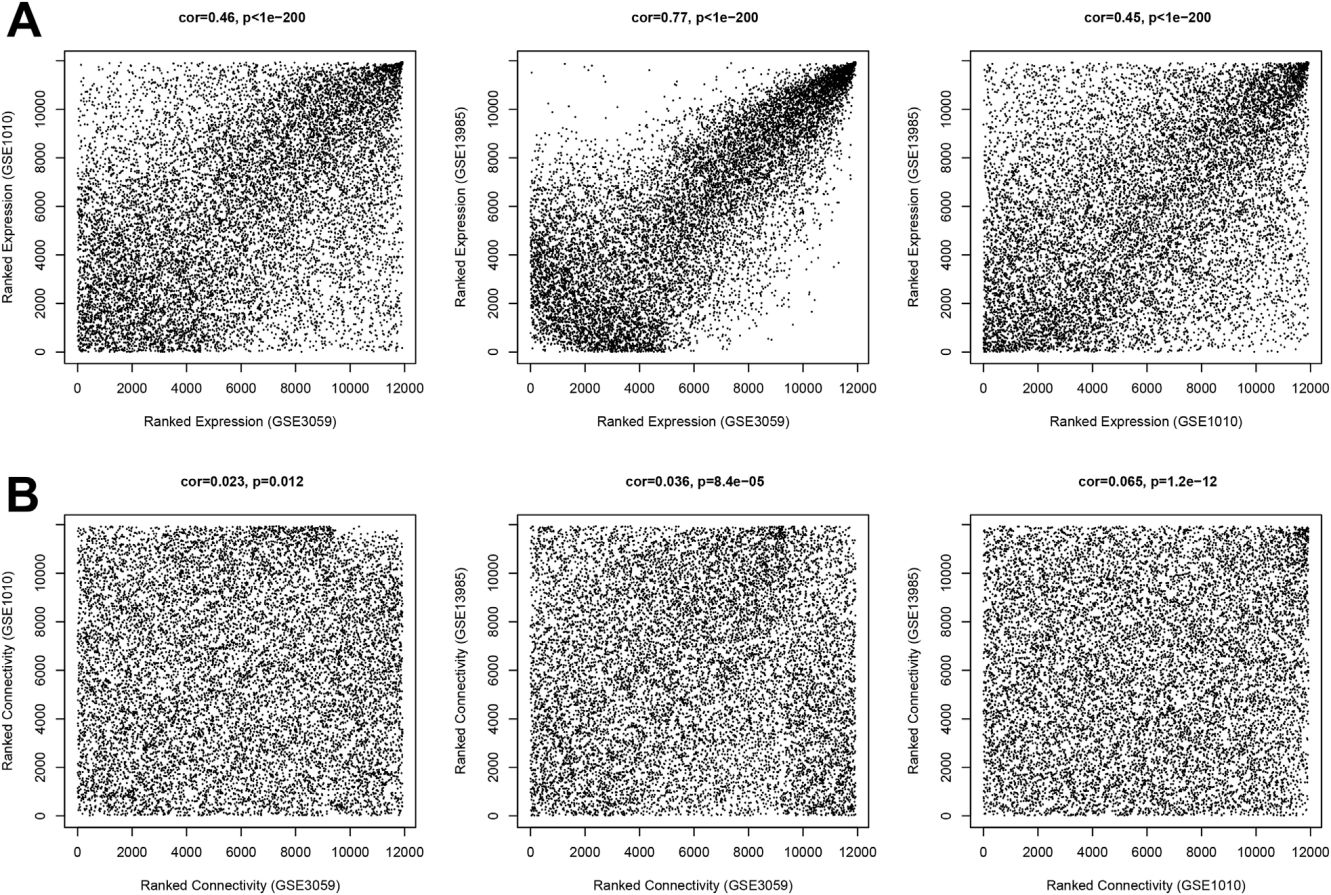
Expression levels of the GSE3095 and GSE13985 GSE1010 datasets. **(A)** Correlation of expression level. **(B)** Correlation of network connection.

**FIGURE 3:**
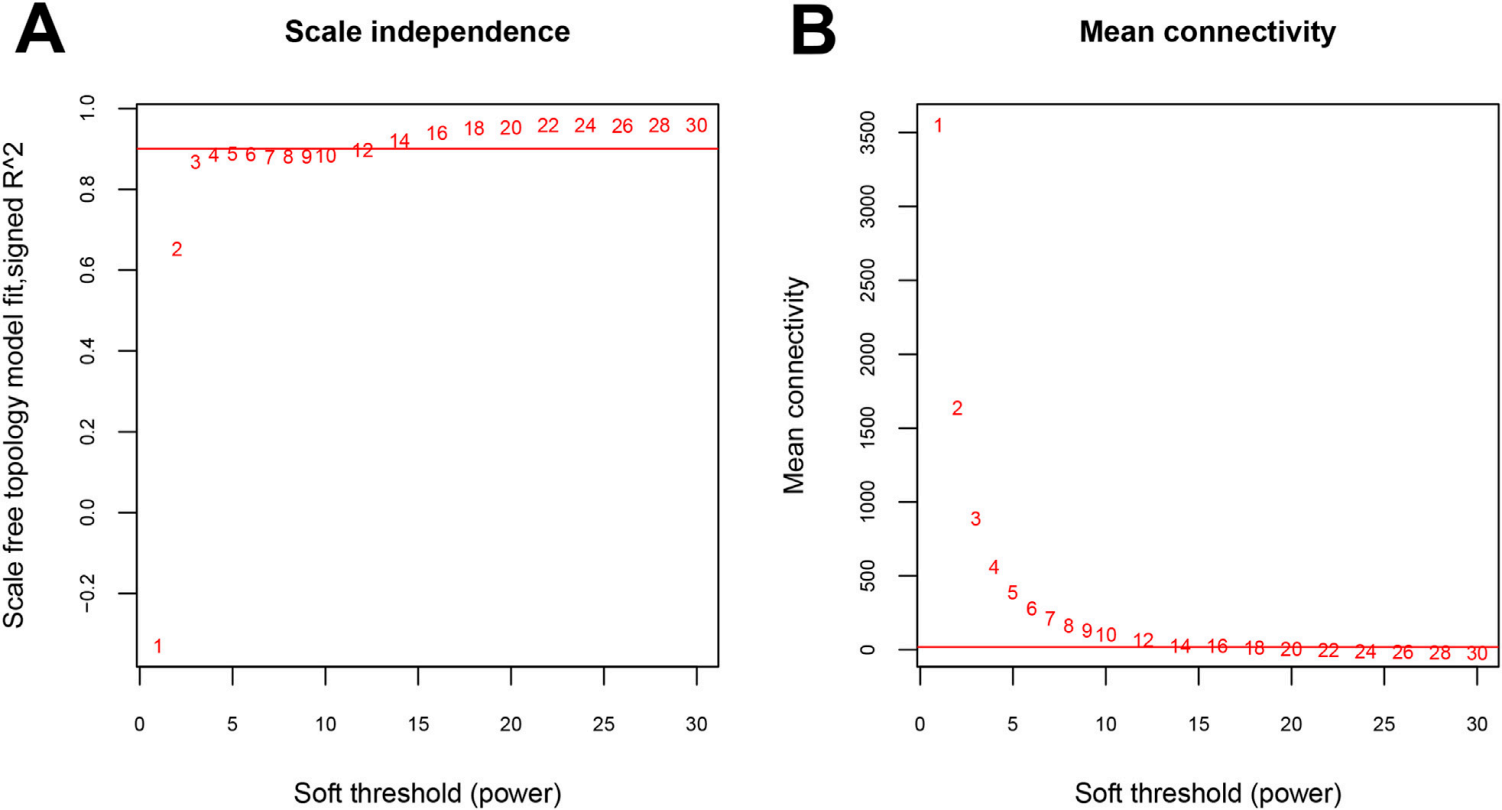
Definition of an adjacency function **(A)** Power selection diagram of the adjacency matrix weight parameters. **(B)** The mean gene connectivity under different power parameters.

**FIGURE 4:**
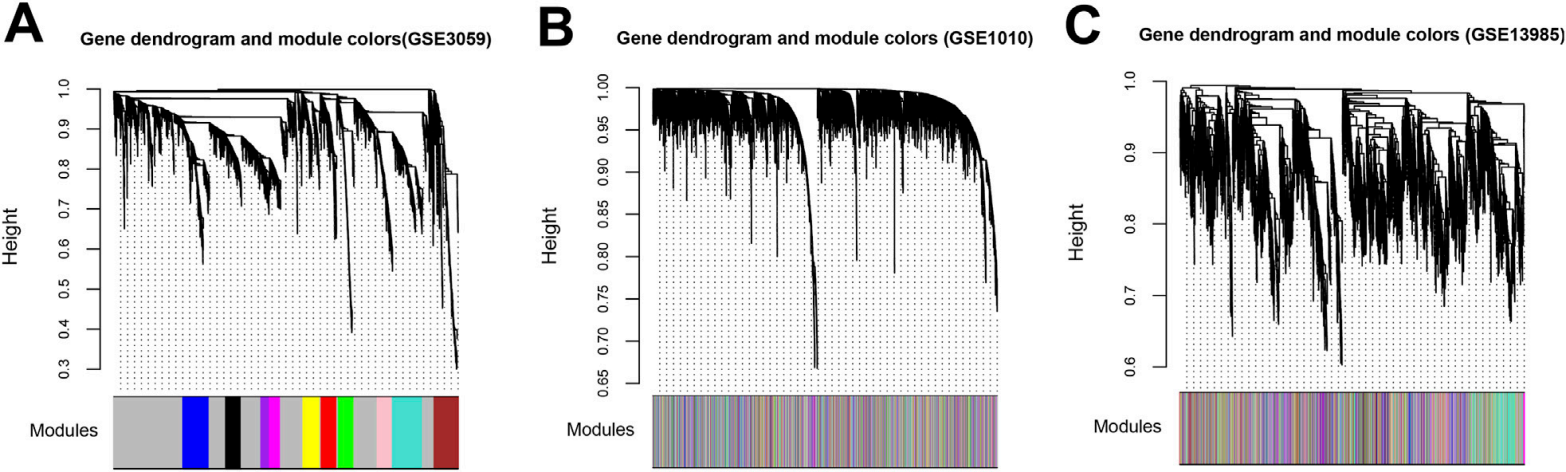
The gene dendrogram of stable gene modules related to hyperlipidemia. **(A)** GSE3095. **(B)** GSE1010. **(C)** GSE13985. Each colour indicates a different module.

GSE3095 was used as a training dataset, while GSE13985 and GSE1010 were used as validation datasets. By calculating the correlations of gene expression levels and network connections between each pair of datasets, it can be observed that the correlation coefficients of gene expression levels ranged from 0.45 to 0.77 (*P* < 1e-200, Figure 2A), and the correlation coefficients of network connections were all greater than 0 (*P* < 0.05, Figure 2B). These results indicate that the common genes between each pair of datasets exhibit good consistency. The square value of the log(k) and log(P (k)) correlation coefficients that corresponded to the different power values were calculated (Figure 3A). Finally, power = 14 was selected. Under the parameter of power = 14, the average connectivity of genes was counted. As shown in Figure 3B, the average connectivity of the genes was one. Moreover, eleven co-expression modules were obtained using WGCNA, indicating that different colours are used in the branches of the system tree diagram (Figure 4). We first extracted the common genes from the three datasets, used the WGCNA method to partition gene modules in the training set, and then evaluated the consistency of these modules across the three datasets via preservation analysis (Table 1). The results showed that four modules (black, magenta, purple and turquoise) had preservation Z scores >5 and *P-*value <0.05. Therefore, the 983 genes in the 4 modules were regarded as an important gene set.

**TABLE 1:** Preservation information of eleven co-expression modules in GSE3095, GSE13985, and GSE1010 datasets.

ID	Color	Number of genes in module				Preservation	
		Training set	Testing set		Replicated genes		
		GSE3095	GSE13985	GSE1010	From training to testing	Z-score	P value
Module 1	Black	235	235	235	235	**10.3079**	7.50E-12
Module 2	Blue	400	400	400	400	2.0745	2.90E-06
Module 3	Brown	359	359	359	359	3.8661	6.10E-21
Module 4	Green	236	236	236	236	1.6561	3.70E-02
Module 5	Grey	2,478	2,478	2,478	2,478	3.3516	1.30E-08
Module 6	Magenta	168	168	168	168	**7.2370**	1.90E-08
Module 7	Pink	226	226	226	226	0.8031	2.50E-01
Module 8	Purple	127	127	127	127	**11.2637**	4.70E-07
Module 9	Red	236	236	236	236	0.8370	1.30E-01
Module 10	Turquoise	453	453	453	453	**10.5177**	9.30E-04
Module 11	Yellow	256	256	256	256	3.8567	8.90E-11

Bolded values in the Z-score column indicate preservation Z scores > 5.

### Selection of DEGs with significant consistency across datasets

**FIGURE 5:**
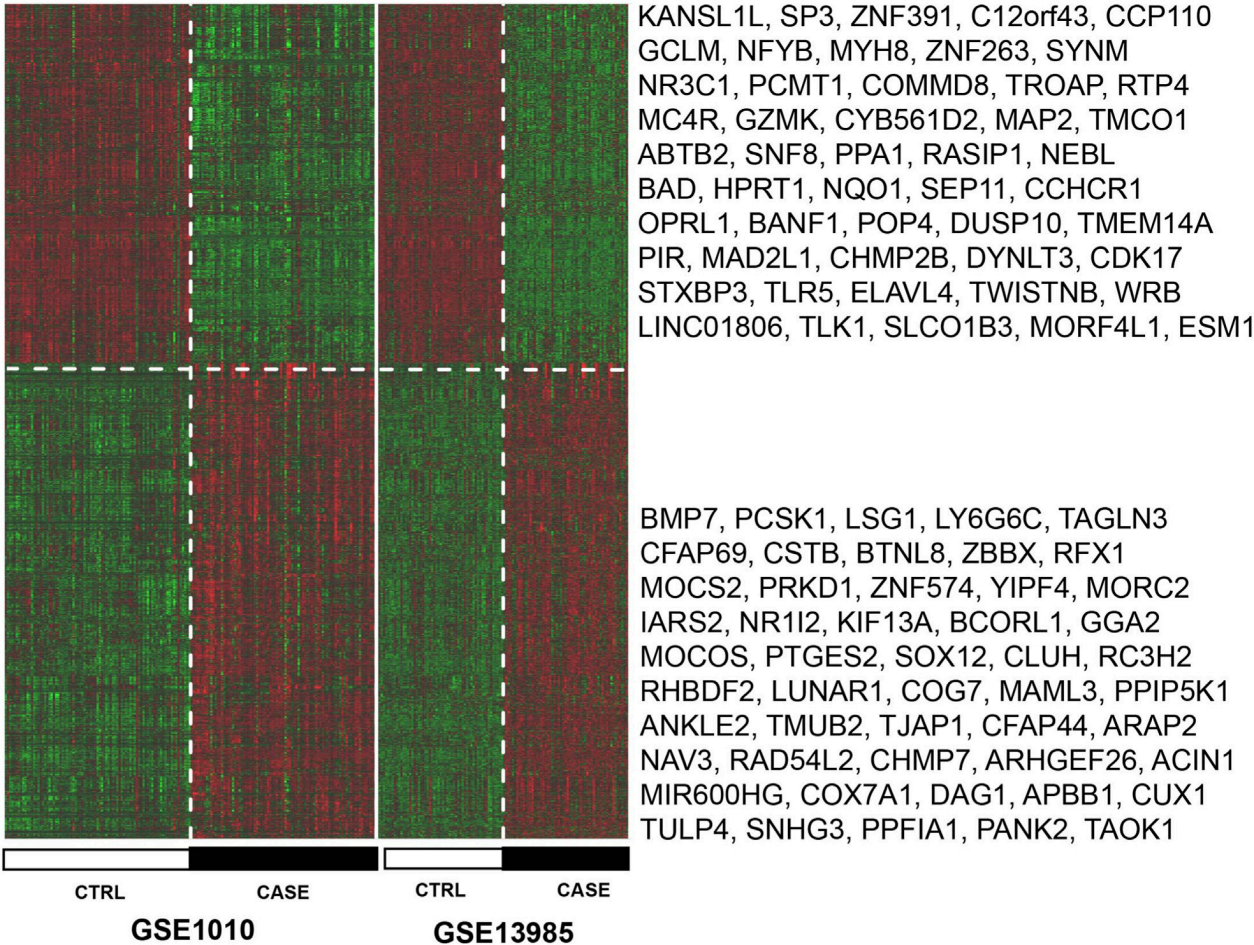
Heat map of DEGs with significant consistency. The black and white sample bars indicate hyperlipidemia and control samples, respectively. The top 50 up-regulated and down-regulated genes with the smallest FDR values have been displayed in the heatmap.

Based on the GSE1010 and GSE13985 datasets, a total of 1,149 genes with significantly different expressions were selected through the comprehensive analysis of the MetaDE package (Supplementary Table S1). Among them, 574 genes were significantly downregulated, and 575 genes were significantly upregulated. At the same time, the heatmap. sig.genes function was used to display the heat map of the DEGs screened. It can be seen from Figure 5 that the DEGs screened from two different datasets have the same degree of difference. However, the expression patterns of the DEGs were different between the hyperlipidemia and control samples.

### Screening of overlapping genes between WGCNA and MetaDE analyses

**FIGURE 6:**
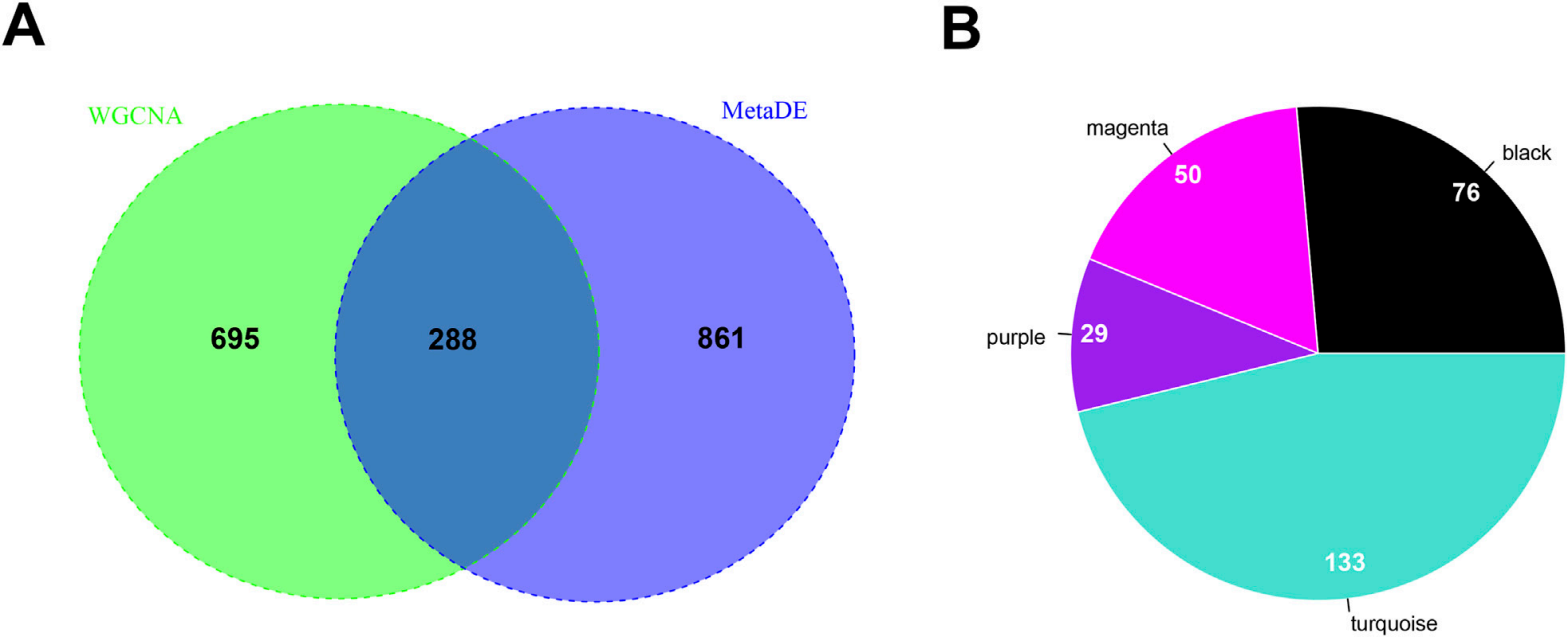
Screening results of overlapping genes based on WGCNA and MetaDE. **(A)** The overlapping genes between the WGCNA and MetaDE. **(B)** The number of overlapping genes distributed in different WGCNA modules. Different colours correspond to different modules in WGCNA, and the numbers indicate the number of genes in the colour modules.

A total of 288 overlapping genes were obtained through comparing the 983 module genes in the 4 significantly stable WGCNA modules with the 1,149 genes with significantly different expressions selected by the MetaDE algorithm, (Figure 6A). Besides, the number of overlapping genes distributed in different highly-conserved gene modules is shown in Figure 6B. The turquoise module includes 133 genes, the purple module includes 29 genes, the magenta module includes 50 genes and the black module includes 76 genes (Figure 6B). Supplementary Table S2 provides the results of GO and KEGG enrichment analyses in the DAVID database for the overlapping genes between WGCNA and MetaDE. Totally, 13 GO annotations and 8 KEGG pathways were selected (Table 2). Significantly related GO annotations included the cytoskeleton organisation, which involved 16 DEGs, such as *RAF1*, *WAS*, *PTK2B* (*P* = 2.669E-03); the ncRNA metabolic process, which involved eleven DEGs, such as *RPF1*, *IARS2*, *PUS7L* (*P* = 2.682E-03) and the actin cytoskeleton organisation, which involved 10 DEGs, such as *ARPC1A*, *PTK2B*, *WAS* (*P* = 7.588E-03). In addition, the natural killer cell-mediated cytotoxicity KEGG pathway involved 6 DEGs, such as *RAF1*, *FCGR3B*, and *PTK2B* (*P* = 1.128E-02).

**TABLE 2:** GO functional and KEGG pathway enrichment analysis of 288 overlapping genes.

Category	Term	Count	P value	Genes
Biology process	GO:0007010∼cytoskeleton organization	16	2.669E-03	RAF1, WAS, PTK2B
	GO:0034660∼ncRNA metabolic process	11	2.682E-03	RPF1, IARS2, PUS7L
	GO:0030036∼actin cytoskeleton organization	10	7.588E-03	ARPC1A, PTK2B, WAS
	GO:0030029∼actin filament-based process	10	1.129E-02	ARPC1A, FMNL1, CORO1A
	GO:0015031∼protein transport	21	1.178E-02	ARL6IP1, CHMP2A, RAB2A
	GO:0045184∼establishment of protein localization	21	1.291E-02	RAB2A, RAB9A, LYN
	GO:0046907∼intracellular transport	18	2.212E-02	TIMM17A, VPS45, WAS
	GO:0012501∼programmed cell death	17	2.362E-02	RAF1, TBRG4, CXCR2
	GO:0008104∼protein localization	22	2.654E-02	RAB2A, RAB9A, LYN
	GO:0006412∼translation	11	2.975E-02	MRPL34, EIF2B4, KARS
	GO:0006915∼apoptosis	16	4.055E-02	RAF1, TBRG4, CXCR2
	GO:0016265∼death	18	4.861E-02	RAF1, TBRG4, CXCR2
	GO:0033554∼cellular response to stress	15	4.933E-02	UPF1, NUAK2, LYN
KEGG pathway	hsa04062:Chemokine signaling pathway	9	2.696E-03	GRK3, RAF1, CXCR2
	hsa04666:Fc gamma R-mediated phagocytosis	6	3.560E-03	LYN,RAF1, WAS
	hsa04144:Endocytosis	8	6.343E-03	TGFBR2, GRK3, CXCR2
	hsa04650:Natural killer cell mediated cytotoxicity	6	1.128E-02	PTK2B, ICAM2, RAF1
	hsa04060:Cytokine-cytokine receptor interaction	9	1.310E-02	IL18R1, TNFRSF10C, IL10RB
	hsa03030:DNA replication	3	1.522E-02	RFC1, PCNA, MCM3
	hsa04110:Cell cycle	5	2.203E-02	HDAC1, CDKN2D, YWHAQ
	hsa04514:Cell adhesion molecules (CAMs)	4	4.705E-02	SELL, ICAM2, ICAM3

### Construction of a PPI network by the overlapping genes

**FIGURE 7:**
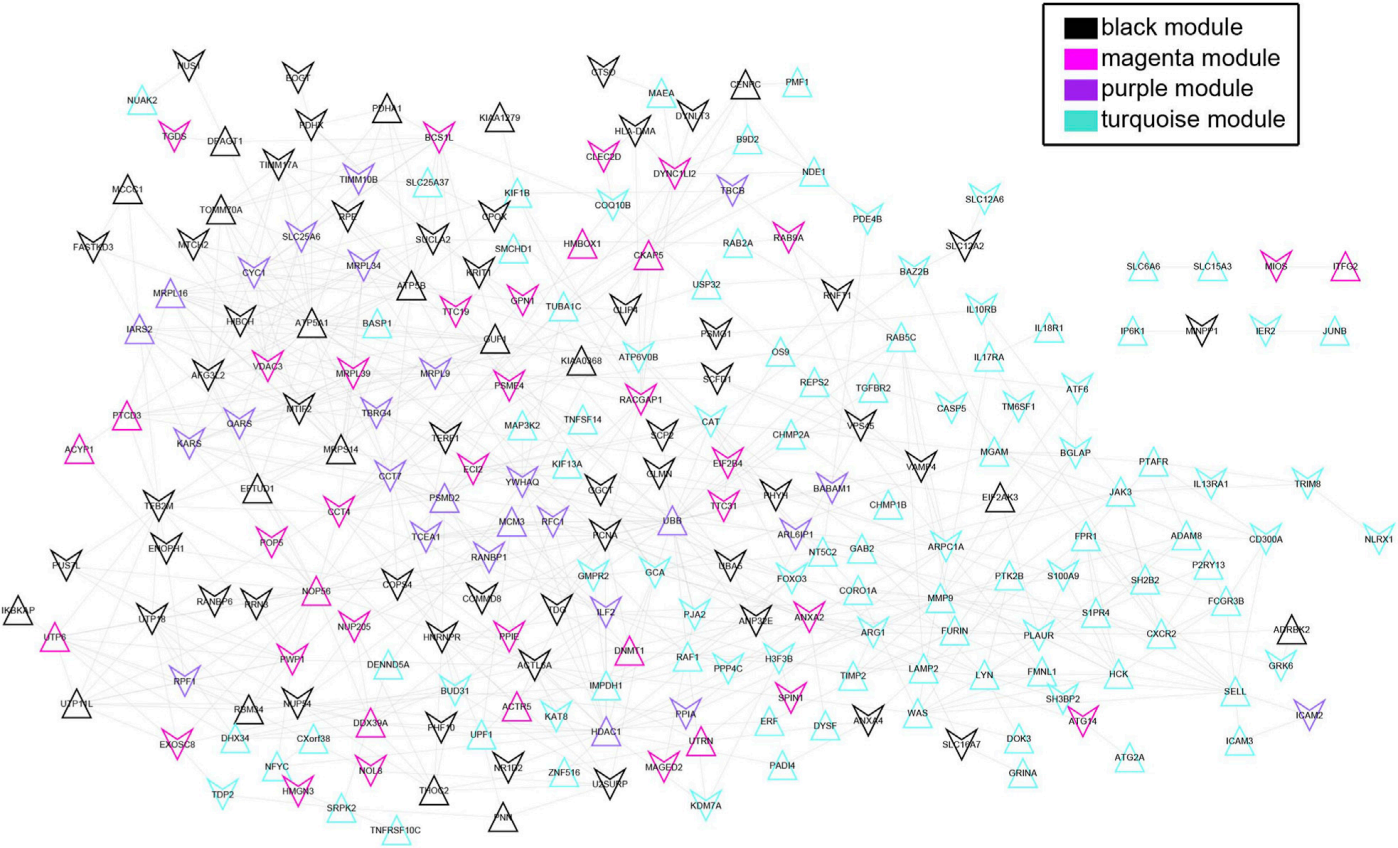
The protein-protein network for the overlapping genes. Triangles and inverted triangles represent upregulated and downregulated genes in the blood tissue of patients with diseases. The colour of nodes indicates the corresponding colour of the gene from the WGCNA module.

Totally, 288 overlapping genes were obtained for PPI network construction (Figure 7). In total, 231 nodes and 628 interactive connection pairs were obtained, including LYN-RAF1, FPR1-CXCR2, GRK6-GRK3, etc. (Supplementary Table S3). A total of four significant KEGG pathways were obtained, including the chemokine signalling pathway, which involved 10 DEGs, including *RAF1*, *GRK3* and *CXCR2*; Fc gamma R-mediated phagocytosis, which involved 6 DEGs, including *RAF1*, *LYN* and *WAS*; endocytosis, which involved 8 DEGs, including *GRK6*, *CXCR2* and *CHMP2A* and natural killer cell-mediated cytotoxicity, which involved 6 DEGs, including *RAF1*, *PTK2B* and *FCGR3B* (Table 3).

**TABLE 3:** The significantly associated KEGG pathways with the feature genes obtained.

Term	Count	P value	Genes
hsa04062:Chemokine signaling pathway	10	2.543E-03	LYN, PTK2B, HCK, GRK6, GRK3, RAF1, CXCR2, JAK3, FOXO3, WAS
hsa04666:Fc gamma R-mediated phagocytosis	6	1.239E-02	ARPC1A, GAB2, LYN, HCK, RAF1, WAS
hsa04144:Endocytosis	8	2.063E-02	CHMP2A, CHMP1B, RAB5C, VPS45, TGFBR2, GRK6, GRK3, CXCR2
hsa04650:Natural killer cell mediated cytotoxicity	6	4.131E-02	TNFRSF10C, PTK2B, ICAM2, RAF1, FCGR3B, SH3BP2

### Screening of feature genes in patients with hyperlipidemia

**FIGURE 8:**
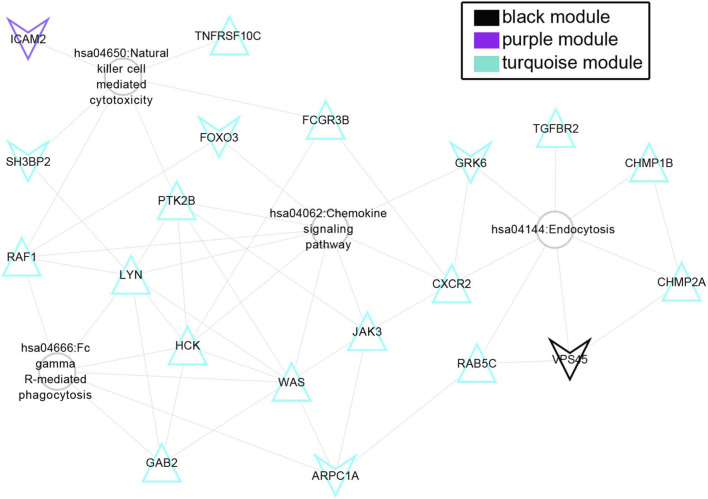
KEGG pathway gene network directly related to hyperlipidemia. Triangles and inverted triangles represent upregulated and downregulated genes in blood tissue of patients with diseases. The colour of nodes indicates the corresponding colour of the gene from the WGCNA module; the round nodes indicate the KEGG pathway directly related to hyperlipidemia.

A total of 77 KEGG pathways were identified to determine the relationship with hyperlipidemia in the searching CTD database. After comparing the KEGG signalling pathways significantly correlated with Table 2, a total of four overlapping pathways were obtained (Table 3). As shown in Figure 8, the four KEGG pathways contain 21 genes, including the chemokine signalling pathway (*LYN*, *PTK2B*, *HCK*, *GRK6*, *GRK3*, *RAF1*, *CXCR2*, *JAK3*, *FOXO3*, *WAS*), Fc gamma R-mediated phagocytosis (*ARPC1A*, *GAB2*, *LYN*, *HCK*, *RAF1*, *WAS*), endocytosis (*CHMP2A*, *CHMP1B*, *RAB5C*, *VPS45*, *TGFBR2*, *GRK6*, *GRK3*, *CXCR2*) and natural killer cell–mediated cytotoxicity (*TNFRSF10C*, *PTK2B*, *ICAM2*, *RAF1*, *FCGR3B*, *SH3BP2*). The Cytohubba plugin in Cytoscape was used to identify the top 5 nodes with the highest Maximal Clique Centrality as hub nodes, including 4 genes (*LYN*, *HCK*, *WAS*, *PTK2B*) (Supplementary Figure S1A). Additionally, the TRUUST database (https://www.grnpedia.org/trrust/) was employed to search for transcription factors corresponding to these hub genes. However, transcription factors were only identified for *WAS* and *HCK*, with *SP1*, *ETS1*, *MYB*, *SPI1*, *RARA*, and *PML* acting as transcription factors (Supplementary Figure S1A). Therefore, the current study suggests that genes involved in these directly related hyperlipidemia pathways such as *RAF1*, *GRK3* and *CXCR2* were significant feature genes associated with hyperlipidemia.

## Discussion

Hyperlipidemia is a complex and multifactorial condition. Its main predisposing factors include genetics, diet, diabetes and obesity. Through a comprehensive analysis of datasets containing hyperlipidemia and healthy control samples, the underlying pathogenesis of hyperlipidemia was explored in this study. Using the WGCNA, eleven highly-preserved modules were detected. Using the metaDE method, 1,149 genes with significant consistency were selected from three datasets. It is worth noting that there were four significantly-correlated enrichment KEGG pathways in the PPI network; these include the chemokine signalling pathway, which involved 10 DEGs, including *RAF1*, *GRK3* and *CXCR2*; Fc gamma R-mediated phagocytosis, which involved 6 DEGs, including *RAF1*, *LYN* and *WAS*; endocytosis, which involved 8 DEGs, including *GRK3*, *CXCR2* and *GRK6* and natural killer cell-mediated cytotoxicity, which involved 6 DEGs, including *RAF1*, *PTK2B* and *FCGR3B*.

*HCK* and *LYN* belong to the Src tyrosine kinase family expressed in neutrophils. Previous pharmacological studies have also indicated that tyrosine kinases may participate in GPCR signaling in neutrophils (Mócsai et al., 1997; Futosi et al., 2013). Miki et al. found that *LYN* plays a crucial role in lipid metabolism under high-fat diet conditions and the development of atherosclerotic lesions (Miki et al., 2001). Prior research has demonstrated that the low-density lipoprotein receptor is essential for clearing atherosclerosis-promoting circulating low-density lipoprotein cholesterol. *WAS* has been shown to be vital for the endosomal sorting and function of low-density lipoprotein receptor (Bartuzi et al., 2016). *PTK2B*, a member of the focal adhesion kinase family, has evidence suggesting its involvement in cell growth, vasoconstriction, and inflammatory responses through the activation of angiotensin II type 1 receptors (Kamide et al., 2007). In this study, *HCK*, *LYN*, *WAS*, and *PTK2B* were identified as hub genes using Maximal Clique Centrality, holding significant implications for future research on hyperlipidemia. They also provide potential genetic biomarkers for the early diagnosis of hyperlipidemia.

RAF1 is the key component of the Ras–RAF–MAPK pathway. It is located in the central position and transmits the signal of activated Ras protein to ERK through MEK kinase (Yang et al., 2020). Some studies have demonstrated that *RAF1* is related to cell proliferation, immunity, tumorigenesis and angiogenesis (Noeparast et al., 2019; Tian et al., 2018; Harms et al., 2018; Jiang et al., 2017). *GRK3*, also known as *BARK2* and *ADRBK2*, is widely expressed in fat. It participates in protein amino acid phosphorylation and signal transduction. Previous studies have indicated that *GRK3* can inhibit the proliferation of tumour cells and smooth muscle contraction (Liu et al., 2018; Billard et al., 2016; Li et al., 2014; Yu et al., 2018). The research results of Wang et al. demonstrated that the high expression of GRKs plays a renal protective role in diabetic nephropathy rat models (Wang et al., 2013). The current study indicated that GRK3 was associated with the chemokine cell pathway and endocytosis. Therefore, based on previous studies, it can be speculated that GRK3 is related to the pathogenesis of hyperlipidemia. CXCR2 is a variety of chemokine receptors, which is closely associated with the inflammatory immune process of the body (Zhang et al., 2019). A previous study showed that the tumour cells expressing CXCR2 drive vascular mimicry in antiangiogenic glioblastoma (Angara et al., 2018). Previous studies demonstrated that *CXCR2* could promote the recruitment of neutrophils in hyperlipidemic mice and is associated with early atherosclerosis (Herz et al., 2015; Drechsler et al., 2010; Bot et al., 2007). The findings of the present study showed that *CXCR2* was associated with the chemokine cell pathway and natural killer cell toxic activity. Thus, it can be speculated that *CXCR2* is closely associated with the pathogenesis of hyperlipidemia and as a biomarker of hyperlipidemia in further research.

Although a comprehensive analysis using three hyperlipidemia datasets was conducted, the study still had many limitations. There is no functional verification of the characteristic genes obtained in this study. To confirm these hypotheses, further investigation of these genes through substantial experiments is required. In addition, the three datasets selected in this study were not from the same platform, and sample stratification analysis has not been carried out, which might have skewed the screening results.

## Conclusions

In summary, this work revealed that the chemokine signalling pathway, endocytosis, Fc gamma R-mediated phagocytosis and natural killer cell-mediated cytotoxicity are key factors in the development of hyperlipidemia. At the same time, the feature genes, including *RAF1*, *GRK3* and *CXCR2* may be potential genetic biomarkers of hyperlipidemia. Perhaps they play a role in hyperlipidemia through multiple pathways. These results can help us further clarify the mechanism of hyperlipidemia and provide underlying genetic biomarkers for the early diagnosis of hyperlipidemia.

## Data Availability

The original contributions presented in the study are publicly available. This data can be found in the Gene Expression Omnibus database with accession number GSE3059, GSE1010 and GSE13985. Further inquiries can be directed to the corresponding author.

## Supplementary material

SUPPLEMENTARY FIGURE S1 Identification of hub genes and their transcription factors. **(A)** Hub genes. Redder colors indicate higher Maximal Clique Centrality scores. **(B)** Transcription factors of hub genes. Blue circles represent transcription factors, and orange squares represent target genes.
