# Supplementary figures and images for: Hyperlipidemia-associated specific modules and hub genes revealed by integrative methods of WGCNA and MetaDE
Source: Front Genet. 2025 Dec 10;16:1592778. doi: 10.3389/fgene.2025.1592778 (PMC12726586; doi:10.3389/fgene.2025.1592778)

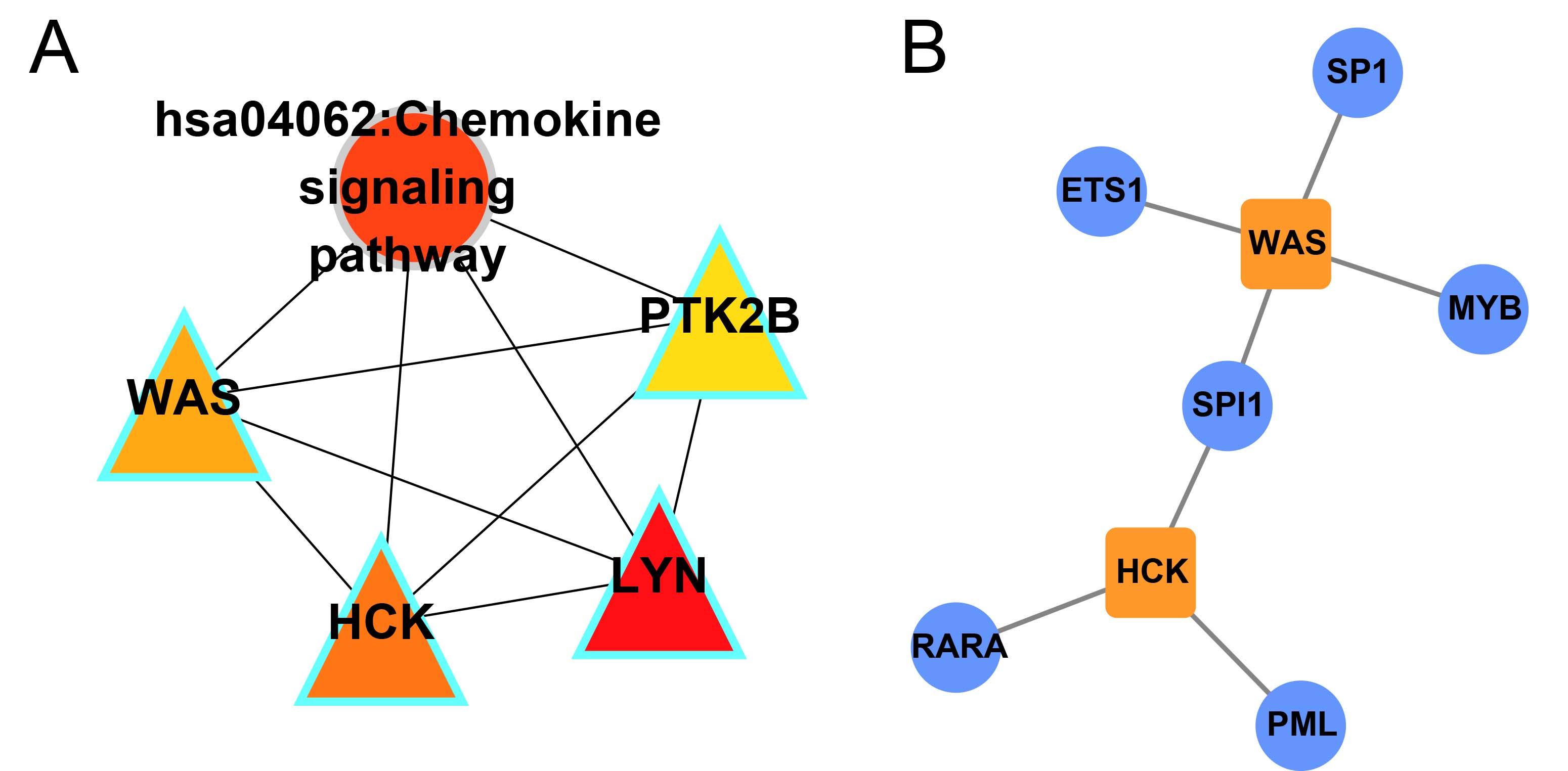

Supplement: Supplementary file 3 [file Image1.jpeg]
